# Supplementary material for: Are carbon emissions trading and green financial instruments synergistic? -Comprehensive quantitative research based on content analysis
Source: PLoS One. 2024 Mar 7;19(3):e0298601. doi: 10.1371/journal.pone.0298601 (PMC10919601; doi:10.1371/journal.pone.0298601)
Supplement: S1 Data — (ZIP) [file pone.0298601.s001.zip › Data/地级市Z_F工作报告.zip!/╜¡╦╒/┴1⁄4╘╞╕█╩╨/└┤╫╘╣1⁄2╓┌║┼í╛┬φ┐╦╩2╛▌═°í┐_2004.docx]

各位代表：

　　现在，我代表市人民政府向大会作工作报告，请予审议，并请各位政协委员和其他列席人员提出意见。

　　2003年工作回顾

　　2003年是本届政府的开局之年，是我市经济社会保持健康发展、呈现积极变化的一年。全市人民在中共连云港市委的领导下，以邓小平理论和三个代表重要思想为指导，认真落实党的十六大和十六届三中全会精神，坚持把发展作为第一要务，克服非典疫情和自然灾害的严重影响，万众一心，奋力拚搏，顺利完成了市十一届人大一次会议确定的各项任务。

　　一、结构调整持续推进，经济质量有效改善

　　过去一年，全市国民经济持续健康协调发展。按省新调整口径初步核算，完成国内生产总值351.1亿元，增长12.1％，创近五年最高增速，一、二、三产业分别增长4％、16.6％、12.8％。三次产业结构调整为23.6∶42.2∶34.2。完成财政总收入34.1亿元，增长26.5％，其中一般预算收入15.7亿元，增长18.2％。财政总收入占国内生产总值比重达9.7％，提高1.1个百分点。

　　全力推进工业强市，主体经济地位快速提升。东陇海产业带规划建设，临港开发正式启动，益海粮油二期工程基本建成、三期油化工项目得到落实，榆嘉制药、亚邦化工等一批临海特色型项目进展迅速，一些大型临港项目正在有序推进。各类工业园区蓬勃发展，在建过千万元的180个工业项目80％以上进入园区，投资过亿元项目18个，工业经济加速向优势产业、优势企业和优势区域集聚。60个新增长点项目对工业销售收入和利税贡献率分别为66.1％和72.8％。完成工业投资120亿元，中电华威环氧模塑料、东海硅微粉等20个重点项目建成投产，新电公司2台33万千瓦机组、罗盖特精细化工等30个重点项目全面开工建设。22户重点企业销售收入占规模以上工业比重达40％。质量工作得到新提高，新确认江苏名牌产品24个，长江三角牌纯碱成为我市第一个中国名牌产品。三资企业、私营工业产值分别增长32％和30.5％，高于全市平均水平12个百分点左右。国内外建筑市场不断扩大，建筑业增加值增长14.5％。

　　结构调整和产业化经营推动农业大灾之年稳步发展，实现农业总产值139亿元，增长3.6％。突出特色、壮大规模，优质稻米、无公害蔬菜等十个优势产业培育初见成效。农产品质量建设加快，农畜产品质量监督检验监测中心通过省级认证，东海被认定为江苏首批无公害基地整体推进县，桃林农业科技示范园区通过国家有机食品认证，新增27个省级无公害品牌，花果山牌风鹅等3个产品获绿色食品品牌。农产品出口创汇保持全省前列，地产小麦面粉首次出口。沿海万亩围栏养殖顺利实施，工厂化养殖扩大到10万平方米，完成海州湾人工渔礁一期工程，建成青口一级渔港，赣榆海洋经济开发区获省批准。实施牧业人均增百元工程，大牲畜、特色养殖较快增长。成片造林41.3万亩，是过去五年的总和。年销售收入5000万元以上农业产业化重点龙头企业达33家。农机跨区作业连续第五年被评为全国先进。建设为农服务社50家，创办专业合作社和专业协会11家。

　　现代物流和旅游经济快速发展，现代服务业和传统服务业协调推进。区域物流中心规划建设，港口物流园区启动实施，国际商务大厦开工建设，新苏豫棉花储运公司成为区域棉花储运中心，邮政快速物流网建设全省领先。港口大宗货物进出口大幅上升，港口完成吞吐量3752万吨，集装箱30.1万标箱，分别增长13.1％和46.8％。运输服务体系进一步完善，市县高等级公路和对外快速通道全面形成，市县区5条客运专线及部分公交线路车辆更新，新浦火车站更名为连云港站，民航站移交地方管理，至北京、上海航班实现每日一班。现代流通方式和流通业态不断涌现，汇金世贸广场、中德商业广场、万润商业街的建设形成新型商贸集聚区雏形。实现社会消费品零售总额123.5亿元，增长12％，居民消费价格指数101.6。成功创建中国优秀旅游城市，花果山景区纳入国家旅游黄金周信息预报，举办连云港之夏、西游记文化节。重点景区实施了一批景点建设和配套设施项目。接待国内外游客500万人次，实现旅游收入41亿元，分别增长31.6％和46.4％。房地产市场交易额21.3亿元，增长66.4％。物业管理面积达600万平方米，建成市、区、街、居四级社区服务网络。金融服务不断创新，银企合作深入开展，金融机构各项存贷款余额分别比年初增加44.5亿元和48.1亿元。实现保费收入10.2亿元，增长40.4％。

　　二、改革领域不断拓展，体制创新取得突破

　　三年改革发展目标如期实现，市属工业企业改制基本完成，国有大中型企业改制面达95％，90％中小企业国有集体资本退出，有效重组存量资产11亿元。恒瑞医药完成国有股权转让，燃气公司出让70％股权。非公有制经济比重提高到45％。民营经济快速发展，新增私营企业2153户，注册资本过百万元的企业774户、超千万元的企业85户，涌现了太平洋石英制品等一批骨干企业。企业上市稳步推进，完成港口股份上市申报和如意股份配股申报，正大天晴、德邦兴华、豪森药业通过上市辅导验收。

　　　推进国有资产管理体制改革，国资委、国有资产经营公司、企业三个层次监督管理体系日趋完善。国有资产监管得到加强，市资产经营公司、土地储备中心等投资主体保持良性运作，新组建港口集团、商业资产公司、交通投资公司和东海、灌南、连云等县区资产经营公司，国有资产市场化运营步伐加快。

　　各项配套改革整体推进。事业单位评聘分开和竞争上岗全面推行，100多家单位试行分配制度改革。市级部门预算全面推行，国库集中收付制度加快实施，政府采购制度进一步完善。市场准入和税收扶持政策明显优化，外贸出口退税政策得到落实。实施重大项目联合审批和重点项目审批代办制度，取消、调整审批事项366项。在全省率先建立市招投标中心。商贸流通、土地流转和户籍制度改革稳步实施，医疗卫生药品三项制度改革有序推进。

　　三、招商引资成效显著，对外开放进程加快

　　全力推进招商引资倍增计划"，认真开展日韩招商年活动，积极发挥友城、侨眷资源优势，注重加强对台招商，务实组织东南沿海、陆桥沿线等境内招商，成功承办第五届江苏农业国际合作洽谈会、举办临港产业合作恳谈会和东海水晶节，引进外资内资均实现倍增目标。协议注册外资5.1亿美元，直接利用外资2.1亿美元，内资客方到位资金36.3亿元。重点依托10个市级以上开发园区开展特色招商，各类园区成为招商引资的主要阵地。出口加工区封关运作，临港产业起步区基本具备项目进区条件，硅信息材料产业园、复合材料工业园等专业园区有序推进，苏北首家留学人员创业园挂牌运作。县区开发园区招商力度加大、项目明显增多，成为区域经济重要增长点。

　　对外贸易稳步增长，进出口总额9.3亿美元，其中出口5.6亿美元，增长12％，外贸依存度提高2.3个百分点。出口商品结构不断优化，出口市场进一步扩大，高新技术产品比重占20％，本地产品比重达60％，新增出口国家26个。外贸企业改制步伐加快，新增自营出口企业64家，民营企业占73％。外经工作稳步推进，实现外经营业额1.1亿美元，增长13％，年末在外人数突破1万人。连众、恒瑞、康缘、德邦等企业走出市外兴办项目。海关、检验检疫、海事、边检强化大通关意识，加强协调服务，有效促进了全市开放型经济发展。

　　四、重点项目建设加快，城市带动能力增强

　　着力实施项目大推进，完成全社会固定资产投资208亿元，增长15.2％，实施水利、交通、电力、通讯、社会服务等36项重点项目。小塔山水库除险加固、东盐河整治、大浦河应急治理、烧香河北闸拆建等重点水利工程顺利推进。连徐高速公路全线通车，连盐高速公路开工建设，新建改建国省道146公里。完成港口10万吨级散货泊位水工工程，2个第五代集装箱泊位、10万平方米保税库开工建设。民航候机楼主体竣工。核电站1、2号机组主体设备安装调试，220千伏和500千伏输变电工程全面展开，建成场外核应急指挥中心、核电环境监测中心和地震监测网。双湖、玉带变电所等12项重点电网项目进展顺利。新增电话交换机容量34万门、宽带用户1.3万户，小灵通市县乡镇联网，联通综合通信楼建成，江苏通信和中国卫星通信连云港分公司成立，电子政务快速推进。新一代天气雷达和自动气象监测网建成使用。市新图书馆、淮工体育馆和教学实验综合楼交付使用，新海高中综合训练馆、东方医院门急诊楼主体建成，市妇儿活动中心、新博物馆、新闻中心、广电中心二期、中医院门急诊综合楼开工建设。

　　强化城市规划建设管理，推进城乡统筹协调发展。城市总体规划和市域城镇体系规划修编完成，编制临港产业区等十个专项规划，重点路段和区域详细规划覆盖率100％。投资34.2亿元实施278项城市建设项目，建成区面积扩大到62.6平方公里，城市综合服务功能显著增强。新区路网框架基本形成，市行政中心主楼封顶，小区开发成片推进。集中拆迁房屋80万平方米，住宅开发213万平方米。新建改建新港城大道、海滨大道等36条主次干道，打通龙河路等多条便民道路，新增城市道路48.5公里。新海污水处理厂二期和东部污水处理筹建工程进展顺利，建成市危险废弃物集中处置中心，发展管道燃气用户5212户，实施第二热电厂技术改造，建成人防工程2.4万平方米。新增城市绿地163万平方米，绿化覆盖率28.6％。建成滨河广场等一批亮化工程，新增亮化面积310万平方米。制定实施环境卫生和市容市貌管理办法，建立城市管理综合执法局，加强定点管理与机动管理，逐步形成长效管理机制。四县县城规划建设水平明显提高，旧城改造、新区开发快速推进，县域经济中心辐射带动功能进一步增强，东海县被确定为中国水晶之都。板浦、墩尚等10个重点中心镇建设各具特色。城镇体系进一步完善，城镇化率达到38％。

　　五、科技教育全面进步，社会事业协调发展

　　大力推进科技创新，实施省以上火炬计划项目21个、星火计划项目44个。恒瑞医药继中电华威之后成为苏北第二家863计划成果产业化基地，4个产品被认定为国家级新产品，9个项目列入国家十五重大科技专项和双高一优计划，23个项目列入省高新技术研究和重点技术创新计划，新增省以上高新技术企业6家，石英基础材料工程技术研究中心成为省级中心，我市被批准为苏北唯一的省制造业信息化示范城市。高新技术产业增加值比重提高到19％。启动与清华、东南大学全面合作协议，实施百名教授科技兴百村工程。积极开展社会科学研究，组建省社科院连云港分院，《新经济》被评为全国十佳省市内部资料性出版物。各类教育加快发展，素质教育不断推进。新建扩建中小学校舍37.6万平方米，调整农村中小学873所，高中段入学率达76.7％。创建1个省级农科教示范基地、1家省级社区教育示范区。引导发展社会力量办学，南洋国际学校建成开学。完成现代教育园区规划。省农垦、盐业公司和铁道部门的学校移交地方管理。引进本科以上非师范类毕业生1154人，公开选拔任用94名领导干部，引进国外智力项目20个，初步形成人才集聚的良好氛围。

　　文化、广电、卫生、体育等各项社会事业健康发展。女子民乐团成功出访欧洲五国，市少儿图书馆对外开放，广场、社区和全民读书月等文化活动蓬勃开展，文物保护得到重视，文化市场健康有序。实施广播发射台整体搬迁，形成电视移动直播能力，新增有线电视用户2.6万户，行政村有线电视联网率达42％。市一院与北京阜外医院合作成立心脏病防治中心，盐业总医院并入市二院。建立严密防控网络，沉着应对，措施果断，夺取抗非典阶段性胜利。传染病发病率低于全省平均水平。成功承办世界女子沙滩排球赛、全国帆船锦标赛等重大赛事，获得省以上体育竞赛奖牌118枚、金牌39枚，举办全国拔河比赛、沙滩秧歌大赛等大型群众体育活动，新建6个全民健身工程和66个全民健身点。市社会福利院通过国际标准质量管理体系认证。统计信息咨询监督职能较好发挥。外事、侨务、对台工作积极服务经济建设，民族宗教、新闻出版、档案、地方志事业稳步发展。

　　可持续发展协调推进。计划生育十五创建活动全面开展，人口自然增长率控制在5.5‰，东海县被确定为全国关爱女孩行动工程试点县。国土资源得到有效保护，复垦整理土地2万亩，编制实施矿产资源总体规划，云台山封山育林8.7万亩，海域功能区划获省政府批准。强化环境综合整治，市区空气质量优良天数达307天，蔷薇河饮用水源水质达标率100％。

　　六、精神文明建设不断深入，民主法制建设得到加强

　　文明行业、文明单位、文明村镇等创建活动进一步开展，获得省精神文明建设五个一工程组织奖。抗非典型耿文灿精神得到重点宣扬,6000元现象宣传讨论促进了社会风气转变，文明诚信、关爱服务等活动广泛开展，陇海步行街被命名为省百城万店无假货示范街，社会文明程度和市民素质不断提高。推荐奥运吉祥物、神奇浪漫之都巡回摄影展、中央电视台《西游记》专题片等宣传推介活动，扩大了连云港的海内外影响，城市知名度美誉度明显提高。国防教育和国防后备力量建设得到加强，双拥工作社会化有序推进，连续第四次获国家双拥模范城称号。

　　积极推进依法治市，依法行政水平进一步提高。四五普法教育深入开展，政府工作人员法律意识和法制观念不断增强。落实行政执法责任制，强化行政执法监督和执法过错责任追究，完成执法主体资格审查认证，各种违法违纪和损害发展软环境行为受到严肃查处。政务公开全面推行，多渠道向社会公布规范性文件，实行公开办事和收费公示制度，落实服务承诺和首问责任制。城乡基层民主建设得到加强，村级规范化建设逐步推开。纠风治乱和行风评议活动深入开展，部门和行业风气不断好转。定期向人大报告工作，主动向政协通报情况，办理代表建议和委员提案624件，广泛听取社会各界意见，政府决策机制逐步完善。

　　健全社会治安防控体系，落实综合治理各项措施，严厉打击法轮功邪教组织和各种违法犯罪活动，深入开展打黑除恶专项斗争，大力推进平安连云港建设，政治大局稳定，治安状况好转。高度重视信访工作，认真解决群众反映的问题，妥善化解各种矛盾，社会保持稳定。坚持从严治政，强化廉洁从政，加强审计监督，全面落实党风廉政建设责任制，深入开展反腐败斗争，认真查办大案要案，一批违规违纪干部受到严肃查处。开展药品、食品、农资专项打假，市场秩序进一步规范。

　　七、生活水平稳步提高，社会保障继续加强

　　坚持把改善民生摆在突出位置，居民生活水平稳步提高。城镇居民人均可支配收入7780元，增长11.9％，农民人均纯收入3230元，增长8％。人均储蓄增加523元。城乡居民恩格尔系数下降1个百分点。电话普及率达每百人30部。归集城镇职工住房公积金2.5亿元，建设经济适用房26万平方米，130户特困户住上廉租房。全面实施农村七件实事。改造草危房9588户，帮助灾民建房4635户。新增改水受益人口31万人。建成农村道路707公里。新型农村合作医疗人口覆盖率达35％。取消农林特产税，减免农业税，实行涉农收费公示制度，农民负担监督卡入户率99.4％。四级联动帮扶到户4.5万户。完成农业综合开发投资7626万元。扶贫开发和老区建设投入资金1.5亿元。

　　实施积极的就业政策，推进生活保障向就业保障转变，成立特困群体再就业援助中心，创建再就业一条街等再就业工程，下岗失业人员实现就业再就业2万人，下岗职工再就业率87％。多渠道输出农村劳动力68.6万人，成为农民增收重要渠道。加大社会保障财政投入，依法扩大社会保障覆盖面，发放离退休人员养老金4.9亿元、下岗职工基本生活费378万元、失业保险金5800万元，投入城市居民最低生活保障金1090万元。医疗、工伤、生育保险覆盖面不断扩大。实施农村居民最低生活保障制度试点。安全生产形势平稳，安全事故起数和伤亡人数实现双下降。

　　各位代表，过去一年的成绩来之不易。这是全市人民在中共连云港市委的领导下，不断解放思想、开拓进取的结果，是方方面面通力合作、共同奋斗的结果。在此，我代表市人民政府，向全市广大干部群众、驻连部队、武警官兵、公安干警、驻连部省属单位和支持连云港发展的海内外朋友，向各位人大代表、政协委员和各民主党派、工商联、人民团体、各界友好人士，表示崇高的敬意和衷心的感谢！

　　回顾过去一年的政府工作，我们有以下几个方面的体会：

　　一是必须强力推进思想解放。思想解放的程度是地区发展的第一成本，思想解放越深刻越到位，发展成本越低、发展步伐越快。过去一年，在工业企业和社会公共产品国有股权转让、吸纳外来资源等方面勇于突破，初步建立人才、土地、资本等要素集聚的政策新机制，进一步夯实了全市上下聚精会神搞建设、一心一意谋发展的思想基础，激发了创业活力，增强了发展动力。

　　二是必须不断创新发展思路。积极探索具有时代特征和自身特色的新途径，是加快发展的基本前提。过去一年，为优化工业布局促进集聚发展，全市注重特色项目向专业园区集中、重大项目向临港地带集中；为有效经营城市，适时组建多元化投资主体促进城市大开发大建设；为推进临港开发，坚持实施东部城区一体化发展，推动港口与城市全面融合，开创了快速发展的新局面。

　　三是必须着力营造发展氛围。抓住时机营造乘势而上氛围，是释放生产力实现快速崛起的必备条件。过去一年，全市深入开展发展项目大推进、服务环境大建设活动，组织实施东陇海产业带建设和临港产业大开发，强力推进招商引资倍增计划，着力强化重大事项督查督办，全力打造投资成本洼地、客商兴业热土，在全市上下形成了比学赶超求发展的浓厚氛围。

　　　四是必须狠抓关键发展措施。把握主要矛盾、实现重点突破，是做好工作推动发展的有效方法。过去一年，全市以项目化推进为重点，确保各项发展目标全面完成；以国企改革攻坚克难为突破，促进非公有制经济快速发展；以宣传推介为手段，打响中国优秀旅游城市品牌；以结构调整、科技兴农、劳务输出等为途径，增加农民收入。通过重点突破推动经济社会全面发展。

　　五是必须强化政府服务职能。加强政府自身建设、提高行政效率效能，是适应新形势新任务的必然要求。过去一年，为广揽中外客商前来投资兴业，各级政府不断强化服务意识，改进服务手段，深化行政审批制度改革，积极为市场主体服务；为切实改善民生，着力实施七件实事改善农村生产生活条件，大力推进再就业工程解决城镇下岗职工再就业问题，努力体现执政为民宗旨。

　　在肯定成绩的同时，我们也清醒地看到，经济和社会发展中还存在不少困难和问题，工作中还有许多薄弱环节：经济总量仍然偏小，县域经济基础比较薄弱；经济总体竞争力不强，财政收支矛盾仍然突出；工业发展还缺少大项目支撑，农业结构调整需要进一步推进；劳动力供大于求矛盾没有根本缓解，社会保障体系有待进一步完善；发展环境需要持续改善，政府职能有待继续转变，等等。对于这些问题，我们将在今后的工作中采取有力措施，切实加以解决。

　　2004年工作任务

　　2004年是认真落实十六届三中全会精神、全面推进新一轮改革的重要一年，是大力实施东陇海产业带规划建设、兴起大开发大发展热潮、提升区域整体竞争力的关键之年。我们必须高举发展第一、实干兴业旗帜，坚持科学态度，发扬务实精神，把握机遇，打破常规，落实超常措施，乘势而上，大有作为，取得实实在在的超常发展业绩。今年政府工作的总体要求是：以邓小平理论和三个代表重要思想为指导，认真贯彻党的十六大、十六届三中全会和省委十届六次全会精神，按照市委九届三次全会部署，以富民强市、快速崛起，全面建设小康社会总揽全局，全面落实以工兴港、以港兴市、以市带农的策略方针，进一步解放思想、抢抓机遇，进一步深化改革、扩大开放，大力实施临港开发，大力调整经济结构，切实壮大非公有制经济，加快培育特色经济和新的增长点，坚持城乡统筹，坚持以人为本，促进经济社会全面、协调、可持续发展。

　　2004年国民经济和社会发展主要预期目标是：国内生产总值增长11％，一般预算收入增长11％，全社会固定资产投资增长15％，社会消费品零售总额增长12％，外贸出口增长12％，直接利用外资增长30％，城镇居民人均可支配收入增长8％，农民人均纯收入增长8％，城镇登记失业率控制在4.7％左右，人口自然增长率控制在6‰以内，城镇化率40％以上。

　　围绕上述目标和要求，着重抓好以下十个方面工作：

　　一、全面推进东陇海产业带开发建设

　　以临港大开发为重点，形成东陇海产业带全面开发建设局面。调整优化产业布局，统筹区域共同发展，加快建设临港产业区和灌河口工业带、海洋经济开发区、沿陇海线制造业基地。积极争取更多基础产业和基础设施项目列入国家和省计划，有效打造国内外产业转移的承接平台，以园区开发为重点推进临港沿线地带企业集群和产业集聚，新上投资过千万元工业项目90％以上进入园区。大力实施临港开发，完成12平方公里临港产业起步区基础设施建设，进一步向西规划拓展，逐步形成冶金、化工、机械等产业集聚区，成为区域经济的增长块。

　　以大力实施以港兴市主战略、提升桥头堡综合功能为动力，推动临港产业快速崛起。按照建设亿吨大港和现代物流中心定位，适应临港产业大发展要求，修编完善港口总体规划，开展临港产业配套岸线规划论证，着手燕尾港、青口港等南北两翼组合港整合规划工作。多元投资加快港口基础设施建设，大力实施港口建设十项工程，建成10万吨级泊位和30号集装箱泊位，加快建设29号泊位，实施墟沟航道扩建工程。推进庙三突堤、10万吨级以上深水航道、东港区20万吨级矿石码头和前三岛30万吨级原油码头等项目前期工作。完善提升港口集疏运功能，加快建设以港口为龙头的现代物流中心。改善港口仓储运输条件，增辟海上运输航线，建设10万平方米保税仓库、10万吨棉花储备库。构筑港口集疏运快速通道，加快建设连盐高速公路，开工建设242省道，新建改建323、236省道部分路段，改造新牛公路，建设机场路，增加至北京、上海民航航班密度，力争开通至海口、哈尔滨航线，继续做好沿海铁路前期工作。加快建设连云港国际商务区，吸引跨国集团组建物流企业和中介机构，加快物流园区和各类市场建设，初步建成功能健全、配套完善的物流体系。建设港口物流信息平台，提高口岸服务水平。完成港口吞吐量4200万吨，集装箱运量力争突破50万标箱。

　　二、积极推动经济持续快速健康发展

　　进一步提升工业经济主体地位，工业增加值占GDP比重提高1个百分点。狠抓重点项目，增强带动能力。完成工业投资130亿元，新上投资过千万元技改项目125个，实施40个重点开工和25个重点竣工项目，着力推进25个投资亿元以上重大项目建设。加快培育市场占有率高、竞争力强的大企业，80个主要新增长点项目新增销售收入35亿元、利税3.5亿元，拉动工业销售收入和利税分别增长17个和15个百分点。依托现有重点骨干企业，壮大医药、化工、食品、纺织等优势行业。培育新兴产业，壮大特色优势。大力发展能源产业，推动核电站1号机组按时发电，加快220千伏和500千伏输变电配套工程建设，促进新电公司2台33万千瓦机组加快建设，积极争取核电二期、抽水蓄能电站和临海能源项目。加快提升新医药产业，推进新医药产业基地快速发展，支持恒瑞医药、康缘药业、正大天晴等企业实施一批高新技术产业化项目。切实壮大新材料产业，推动中电华威产业化基地、硅信息材料产业园和复合材料工业园加快建设。提高重点企业生产技术装备水平及检测手段，推动传统产业运用高新技术加快改造升级。进一步做大建筑业，建筑业增加值增长14％。

　　着力发展农业和农村经济、增加农民收入，高度重视解决三农问题。优化优势农产品区域布局，推广优质新品种和高效节本新技术，实施特色化生产、规模化发展，进一步培育壮大优质稻米、无公害蔬菜等优势产业，保持粮食综合生产能力，重点发展特种经济动物、肉牛、肉猪等带动性强的畜牧业，加快发展花卉、苗木等园艺业，进一步推进林业产业化。加快浅海开发，扩大工厂化养殖，大力发展海淡水养殖。推进农业产业化经营，重点扶持金田集团、榆城集团等106家销售收入过千万元规模龙头企业做大做强，推动11家省级龙头企业加快发展。加快推进外向型农业发展，强化农产品质量安全建设，农畜产品质量检测中心建成区域性中心，建设一批有机食品基地，无公害农产品基地发展到170万亩，无公害农产品、绿色食品、有机食品品牌发展到70个。加强农产品与市场对接，搞活农产品流通。面向城市市场发展都市农业，推动城乡经济协调互动。支持农民按照自愿民主原则发展多种形式专业合作组织，鼓励工商企业和社会力量投资发展农产品加工和营销。切实改善农村生产生活条件，继续实施小塔山水库除险加固、烧香河北闸拆建、海堤达标和农村水利建设等工程，农业机械化水平提高到78％，以乡村综合服务社为依托完善农业服务体系。充分挖掘农民增收潜力，大力实施农村生态家园富民工程，千方百计增加农民收入。

　　优化服务业发展规划和产业体系，提高服务业发展水平。整合资源大力发展旅游业，变旅游资源优势为产业优势，提升旅游业整体竞争力。按照中外知名旅游城市定位，修编旅游业发展总体规划，成立旅游信息咨询中心，改善旅游管理体制，规范开发旅游资源，创建孔望山国家AAAA级旅游区。大力开拓国际国内旅游市场，深入发掘《西游记》文化内涵，开展猴文化主题旅游系列活动。开发地方特色旅游商品，举办新亚欧大陆桥旅游品博览交易会。实施旅游资源有偿出让经营，吸引社会资金发展旅游业。接待国内外游客550万人次，实现旅游总收入46亿元。加快发展现代商贸流通业，以南京中央商场、美国普尔斯玛特、泰国正大现代商业进入为契机，重组形成中央百货公司、凯玛特购物中心、易初莲花购物中心等商贸集聚区。改造提升传统生产资料市场，发展农资和工业品连锁经营，加快新型流通业态向农村延伸。大力发展信息产业，推进城域传输骨干网、第三代移动通信、无线市话扩容升级等基础设施建设，启动建设数字连云港。加快发展电子政务、电子商务、宽带接入网和新视通业务，建设万润数码港。引导房地产业健康发展，竣工住宅95万平方米，规范房地产市场，推进物业管理市场化。探索新型社区建设管理机制，鼓励社会力量创办便民社区服务机构。推进银企合作，引导金融机构大力开展信贷营销，支持金融企业改革，把农村信用社改造成服务农村社区的地方性金融企业。促进保险业创新服务。规范发展律师、会计、评估等中介服务业，发挥商会、行业协会等自律性组织作用，组建国家级紫菜行业协会。

　　三、务实推进新一轮体制改革创新

　　以新一轮思想大解放推进和完善改革，攻坚克难破除体制障碍，形成更具活力、更加开放、更有效率的经济体系。大力发展混合所有制经济，促使股份制成为公有制主要实现形式。重点鼓励、支持和引导非公有制经济领先发展，非公有制经济比重提高到55％。从现阶段全市经济格局出发，鼓励支持、放手发展民营经济，大力推动全民创业。注重在产权制度、市场准入、扶持激励等方面勇于突破，充分释放民营经济活力，开辟生产力发展新空间。加快落实现代产权制度，促进产权归属清晰、权责明确、保护严格、流转顺畅，切实保障所有市场主体平等法律地位和发展权利，切实保护私有财产权。进一步放宽市场准入条件，促进非公有资本进入法律法规未禁入的所有行业和领域，清除一切歧视性观念、规定和做法，推动各类经济竞相发展。在投融资、注册、税收、用地、外贸等方面同等对待各类企业，健全中小企业融资担保体系，支持企业做大做强，有效激发创业创新热情。不断培植壮大本地民营企业，大力招引扶持外来民资，促使民众自主创业和企业自主创新成为最活跃的发展因素。

　　全面完成国有企业改制任务，进一步完善国有资产管理体制。加大破产企业资产重组力度，促进资本向优势企业、优质资产和优秀企业家集中，加快优强企业资本扩张。巩固改革成果，有效推进锦屏磷矿、涤纶厂等企业改制。积极筹措改革成本，保障改制企业职工利益，规范处理用人单位劳动用工合同，促进职工依法有序合理流动。加快商贸流通企业改革，完成百货总公司、燃料集团等企业改制任务。扩大企业改革领域，打破行业垄断，推进建筑、旅游、交通、市政公用事业等领域市场化改革。加快企业上市步伐，规范操作港口股份、正大天晴、豪森药业、德邦兴华、中电华威等企业上市工作，争取年内上市1家、申报发行材料3家，新组建股份公司2家。规范政府国有资产管理职能，进一步明确市国资委职能，健全政府投资决策和项目法人约束机制。推进投融资体制改革，有效整合现有13个市级资产经营管理公司，新组建教育、体育、水务投资主体，广泛吸纳社会资金促进各项事业发展。健全国有资产监管体系，建立国有资产经营预算体系，完善国有资产授权经营、保值增值和经营者奖惩考核制度。改革分配制度，建立健全经营者收入分配激励和约束机制。

　　大力推进事业单位改革，分类定性职能，完成行政管理型事业单位改革，合理调整和重新界定职能；加快社会公益型事业单位改革，搞活用人和分配制度；推动生产经营型事业单位整体转为企业，吸纳多元投资转轨改制。完善职称评聘分开制度，事业单位聘用制面达80％。深化财税制度改革，按照简税制、宽税基、低税率、严征管原则，分步实施税制改革。健全公共财政体制，明确各级政府的财政支出责任，深化部门预算、国库集中收付、政府采购和收支两条线管理改革，扩大财政直接收付范围。完善农村经济体制，深化农村土地制度改革，引导农户依法、自愿、有偿流转土地经营权，发展适度规模经营；健全农业社会化服务、农产品市场和对农业的支持保护体系，深化农业科技推广体制和供销社改革，完善粮食生产直接补贴办法保护种粮农民利益；落实户籍制度改革政策，促进城乡人口自由流动。推进医疗卫生药品三项制度改革，深化公共卫生体制改革，建立健全疾病预防控制、突发性公共卫生事件医疗救治、重大疫情信息网络、卫生执法监督等多层次医疗保障体系，培育竞争性医疗卫生服务体系。

　　四、全方位加快开放型经济发展步伐

　　抓住国内外生产要素重组和产业转移重大机遇，发挥东陇海产业带和临港产业区规划预期效应，利用国家级开发区和各类专业园区特色品牌，依靠优秀旅游城市和不断优化的投资环境，发起新一轮招商引资的强劲攻势，形成各种生产要素快速集聚态势。在优质载体招商上求突破。依托港口、交通、能源、桥头堡等特色优势，整合推介可经营变现的基础设施、国有土地、骨干企业、存量资产等有效载体，引进国内外有实力的企业促进产业集聚，提升规模效应。在重大项目招商上求突破，围绕新医药、新材料、资源加工等特色产业，大力引进能够拉长产业链的重大项目，围绕石化、冶金等重大基础产业，力争突破一批基地型、龙头型、科技型大项目。在重点领域招商上求突破，强化工业招商引资主体地位，加大农业、服务业、公共设施、社会事业等领域引资力度，推进文化、教育、体育、卫生等领域开放进程。在重点地区招商上求突破，境外继续主攻日韩、扩大港台、拓展欧美，国内重点突出沪浙闽粤、三线军工，加强与国际国内大型企业合作，有针对性地吸引整体迁建。在投资环境招商上求突破，按照国际惯例、服务客商要求，优化税务关务事务服务，强化项目跟踪服务，排除投资障碍，创建适宜创业、适宜居住的投资环境。

　　适应国家整顿规范开发区的政策导向，充分发挥我市土地适宜、布点合理的比较优势，集中力量迅速推动园区建设乘势而上，快速形成开放型经济主阵地、区域竞争制高点，园区利用外资占全市70％以上。大力提升整体竞争力，加快构建开发区、出口加工区、临港产业区、专业园区功能互补的园区体系，分类推进县区开发园区建设，引导各类园区突出特色、错位竞争。加快提升开发层次，抓好高新技术产业园区、大浦化工区、新医药科技园、复合材料工业园、硅信息材料产业园、纺机工业园等特色园区，加大投资强度，增强创新能力，尽快成为新，工业化先行区。牢固树立项目优先观念，协调推进项目建设与功能开发，以园区开发吸引项目，以项目建设扩张园区，形成快速集聚效应。

　　继续推进市场多元化、以质取胜和科技兴贸，调整优化外贸出口主体和出口商品结构，扩大三资企业和生产企业出口规模，促进工贸联合，引导中小企业和非公有企业扩大出口，提高出口商品质量、档次和附加值，扩大高新技术产品和服务贸易，确保外贸出口较快增长。吸引国内外大公司建立出口生产基地，利用出口加工区大力发展加工贸易。推动口岸管理体制与国际接轨，提高贸易便利程度。落实外贸出口扶持政策，改善外贸经营环境。研究利用与香港商品自由贸易新机遇，加强与香港经贸合作。整顿规范劳务市场秩序，大力组织劳务输出。积极推动有条件企业开展境外投资，参与区域经济合作，拓展发展空间。

　　五、进一步提升区域性中心城市功能

　　按照区域性中心城市定位，遵循城市基本发展规律，以现代理念审视城市，以战略眼光规划城市，以精品意识建设城市，以市场化手段经营城市，不断增强中心城市的产业承载力和对外影响力。围绕创建国家环保模范城市、国家卫生城市、省级文明城市、省级园林城市，统筹规划城市空间，科学布局基础设施、产业发展和人居环境，加快建设具有合理功能分区、先进交通网络、配套设施条件、优良城市秩序的国际性海港城市，做大做优做美中心城市，着力塑造海滨风光、山城相拥、绿色生态、现代港城的城市特色。坚持规划先行，扩大城市规划覆盖率，完善城市总体规划成果，高起点高标准高水平编制各城区分区规划、东部新区详细规划、主要广场节点景观规划。

　　继续加大城市建设力度，投资40亿元实施300项城市建设项目。完善城区路网，进一步拉开大城市发展框架。新建改建世纪大道、人民西路、平山路等40条城市主次干道，新海地区建成一环、打通二环、建设三环，完善配套设施，东部城区建成一环、建设二环，完善道路网络，增强集聚功能。续建新港城大道和海连东路，加快东西城区联接。改造城区水系，营造碧水环绕的城市水环境。打通、美化城市水脉，实施玉带河拓宽整治，完成东盐河整治二期工程、开工建设三期工程。完成新区初步开发，形成城市新区扩张效应。市行政中心主楼建成使用，基本完成路网管线等市政设施，颐和花园、香溢花园等小区开发形成适宜人居的成片住宅群。加快旧城改造，全面提升城市形象。拆迁改造陇海饭店、龙河商城、金桥公司等沿街地段和棚户区，完成旧城拆迁90万平方米，实施住宅平改坡工程，加快建设一批标志性建筑，维修建设民主路、南极北路等一批便民道路。加快公共设施建设，完善城市综合服务功能。建成大浦污水处理厂二期工程，开工建设墟沟污水处理厂，实施供电、供水、供气、供热等一批市政公用项目。开工建设人防应急指挥中心，建成人防工程3万平方米，促进人防向民防转变。推进城市绿化美化，打造绿色生态品牌。新增城市绿化面积500万平方米，其中公共绿地110万平方米。实施一批美化亮化项目，新增亮化面积52万平方米。切实提高城市管理水平，创建整洁、清新、靓丽、文明的城市环境。

　　完善市域城镇体系，加快城镇化进程，统筹城乡发展。进一步强化四县县城规划建设管理，提高建设品位，推进新区开发，增强产业支撑，吸纳集聚人口，提升县域中心功能，加快向中等城市迈进。坚持综合开发、配套建设，因地制宜加快10个重点中心镇发展，发挥在城乡发展中的纽带和牵引作用。大力推进县域经济社会发展，切实增强四县在全市发展中的基础地位，形成中心城市、县城、重点中心镇互促并进、协调发展的良性格局。

　　六、着力增强科技教育人才支撑作用

　　大力推进科技进步和技术创新，不断提高产业科技水平。加快发展特色优势产业，提升国家火炬计划新医药产业基地、中电华威新材料和恒瑞股份新医药国家863计划成果产业化基地研发能力，创建国家级硅材料产业基地，形成国内一流的特色产业创新基地，培育高新技术产业群。实施火炬计划项目20项，培育省高新技术企业3家，开发高新技术产品30个，实施20项制造业信息化示范项目。高新技术产业增加值比重提高到20％。深化产学研合作，挖掘地方院校和在连科研院所科技潜力，落实与清华、东南大学科技合作协议。增强企业自主创新能力，提升重点骨干企业研发水平，培育和发展一批拥有自主知识产权、核心竞争能力强的优势企业和产业。重视发挥社会科学作用，加强对全市发展战略问题的调查研究。启动编制国民经济和社会发展第十一个五年计划。

　　高标准发展各类教育，全面推进素质教育，提升教育发展内涵。巩固和完善县级为主的农村义务教育管理体制，推进城乡教育均衡发展。加快发展高中段教育，高中段入学率达80％。推进教育信息化，建成以教育城域网为中心、辐射全市中小学的教育信息网络。加速发展高等教育，优化资源配置，提升办学层次，开工建设现代教育园区。推进非义务教育投资主体多元化，发展民办职业技术学院，促进学校后勤服务社会化。积极发展中高级职业教育和社区教育，组建职业教育集团。切实加快农村职业教育和成人教育发展，大力培养拥有优秀技能的劳动者，增强就业、创新、创业能力，努力把人口压力转变为人力资源优势。

　　牢固树立人才是第一资源的观念，充分发挥人才在经济和社会发展中的基础性、决定性作用，大力实施人才强市战略。强化人人都可以成才的意识，既重视拔尖人才又重视实用人才，既抓好人才教育培养又抓好人才激励使用，努力营造鼓励人才干事业、支持人才干成事业、帮助人才干好事业的体制机制。制定促进人才成长的政策措施，设立人才队伍建设资金，努力建设一支以党政人才、企业经营管理人才和专业技术人才为主体，结构合理、素质较高的人才队伍。以发展引人才，靠人才促发展，不断开创人才涌现、人尽其才的生动局面，为全面建设小康社会提供人才保证和智力支撑。

　　七、统筹促进各项社会事业共同进步

　　繁荣发展文化艺术，深入打造在海一方文化品牌，做好历史文化名城申报准备工作，建成市新博物馆，新建市文化艺术中心，组织中法文化年秀女乐坊文化交流活动。推进有线广播电视进村入户，开展数字电视播出试验，完成市广电中心二期工程，新中波发射台投入使用。整合资源组建市一院、二院医疗集团，开工建设市一院科技信息楼，建成中医院门急诊综合楼,加快建设东部城区医疗机构，推进公立医疗机构股份制改造。鼓励城市医院帮扶农村卫生机构，全面完成乡镇卫生院改制。实施国民体质测定标准，广泛开展全民健身活动，承办世界男子沙滩排球巡回赛和全国高水平沙滩排球、武术散打比赛，开工建设新体育场。开展第一次经济普查。续修连云港市志。抓好外事、侨务和对台事务。做好物价、档案、气象、地震、新闻出版、民族宗教工作。重视妇女儿童、老龄和残疾人事业，完成市少儿科技活动中心主体工程，加强社会福利和老年服务设施建设。

　　强化人口与计划生育工作，扎实推进十五创建，全面开展技术优质服务，启动计划生育困难家庭救助工程，推广关爱女孩行动工程，计划生育率达89％。加强土地复垦开发，保持耕地总量占补平衡。编制县级矿产资源总体规划，加快推进马涧工程，继续抓好云台山封山育林。组织实施第四轮市长环保目标责任状，启动生态市规划建设，实施蓝天、碧水等八项工程。

　　八、大力加强精神文明和民主法制建设

　　以创建省级文明城市为目标，大力开展文明行业、文明单位、文明村镇等创建活动，落实公民道德建设实施纲要，提高城乡文明程度和市民文明素质。以创建文化大市为目标，加强特色文化建设，推动文化产业发展，继续开展奥运吉祥物推荐活动。以构筑终身教育体系为目标，搞好家庭、机关、社区、企业等学习型组织建设，形成全民学习的浓厚氛围。加强国防教育和民兵预备役建设，成立市拥军协会，申报省级双拥模范县区。

　　认真贯彻人大及其常委会决议，自觉接受人大监督，坚持重大事项报告制度，积极支持政协、民主党派、工商联履行政治协商、民主监督和参政议政职能，认真听取人民团体意见，支持司法机关依法办案。加强基层民主建设，扩大公民有序的政治参与。探索现代企业制度下职工民主管理的有效途径，依法维护职工合法权益。加强社区居民自治组织建设，实施第七届村委会换届选举，全面推进村级规范化建设。广泛开展四五普法教育，增强全民法律意识和法制观念。自觉运用法律手段调节、管理经济社会事务，推进行政行为和行政程序法定化，提高依法行政水平，促进法制城市建设。强化行政监察，推行行政执法评议考核制和执法过错责任追究制，做到严格执法、公正执法、文明执法。

　　强化社会治安综合治理，依法严厉打击各种违法犯罪，建设平安连云港，创建最安全地区。打击骗税逃税、制假售假等违法犯罪行为，加强质量技术监督，整顿规范市场经济秩序。把改革力度、发展速度和社会可承受程度统一起来，高度重视人民来信来访，认真落实信访工作责任制，努力把矛盾化解在萌芽状态、解决在基层，确保社会稳定。深入开展廉政建设和反腐败斗争，认真落实廉政建设责任制，切实加强审计监督，抓好政府工作人员特别是领导干部的廉洁自律，管好自己、管好家人、管好身边工作人员，坚决查处各种违纪违法案件，树立廉明政府形象。建立健全与市场经济体制相适应的教育、制度、监督并重的惩腐倡廉体系，始终坚持两个务必，坚决抵制各种不良风气的侵蚀。

　　九、切实改善和提高城乡人民生活质量

　　正确处理富民与强市的关系，更加重视和关心人民群众生活，使人民群众在改革和发展中得到更多的实惠。推行工资集体协商、工资指导线、最低工资标准制度，注重扩大中等收入者比重，提高低收入者收入水平。集中建设26万平方米经济适用房，抓好廉租房建设。继续实施农村七件实事，进一步改善农村生产生活条件。改造草危房8500户。新增改水受益人口31万人。建设农村公路700公里。新型农村合作医疗以村为单位覆盖率达60％。继续推进农村税费改革，进一步减轻农民负担。推进扶贫开发富民工程，加快老区建设。投入7000万元继续实施农业综合开发。改善农村消费环境，有效启动农村消费市场。

　　把扩大就业放在经济社会发展更加突出的位置，注重发展劳动密集型中小企业和非公有制经济，加快公共事业、社区服务业发展，积极开发就业岗位。落实下岗失业人员再就业优惠政策和城市低保对象收费减免政策。进一步开展再就业援助行动，政府继续出资购买岗位，解决特殊困难群体就业问题。加快建设与经济发展水平相适应的社会保障体系，提高财政对社会保障支出比重，扩大社会保险覆盖面。以社会化管理和可持续发展推进城镇养老保险制度改革，重点解决好外商投资企业、私营企业、个体工商户和自由职业者参保问题，积极发展商业养老。力争实现下岗保障向失业保险全面并轨。合理提高城镇低保水平，切实关心弱势群体和困难群体。逐步降低医疗费用个人负担，健全社会医疗救助体系。积极拓展农村就业空间，逐步统一城乡劳动力市场，为农民创造更多就业机会，加快发展劳务经济，新增劳务输出10万人。在有条件地区建立个人缴费为主、集体补助为辅、国家政策扶持的农村社会养老保险制度，建立健全农村最低生活保障制度。积极完善救灾、互助、福利等社会救助体系。切实保障居民饮食、卫生安全。强化安全生产监管，确保人民生命财产安全。

　　十、与时俱进切实推进政府职能转变

　　面对新的形势新的任务，我们将按照为民、务实、清廉的要求，树立科学的发展观和正确的政绩观，以与时俱进的创新精神，奋发有为的崭新姿态，不断开创新局面，创造新业绩。

　　执政为民建设服务型政府。时刻牢记为民服务宗旨，坚持五个统筹推进全面、协调、可持续发展，以人为本善待群众，加快发展厚待群众。统筹城乡发展重点解决三农问题，统筹区域发展着力推动县域经济崛起，统筹经济社会发展注重解决民生问题，统筹人与自然和谐发展有效保护生态环境，统筹国内发展和对外开放着重引进外来资本。加快推进政府职能转变，市级机关带头转变作风，全面提高行政效率效能和服务管理水平。认真贯彻《行政许可法》，规范政务行为，推行新一轮审批制度改革，努力创造良好发展环境，为市场主体提供优质高效的服务。

　　求真务实建设诚信型政府。视诚实守信为基本行为准则，以身作则取信于民，推动全社会不断增强信用意识，形成以道德为支撑、产权为基础、法律为保障的社会信用制度。信守政府承诺，政府向人代会报告的工作、向社会作出的承诺做到言而有信、一诺千金，提高政府诚信度；实行政务公开，建立科学化民主化规范化的公共决策机制，实行重大决策调查研究、公示听证、专家咨询制度，保障公众知情权，提高政府公信度；履行行政责任，建立严格的绩效考核体系，强化目标管理，加强社会评议和组织考评，保证工作措施到位和目标任务落实，提高政府可信度。

　　开拓创新建设学习型政府。顺应时代潮流，推进公务员尤其是领导干部不断加强理论武装、知识更新和能力培养，着力提高执政为民能力和依法行政水平。注重政治理论学习，深刻领会邓小平理论和三个代表重要思想的精神实质，不断提高思想政治素质，有效增强分析判断能力。注重业务知识学习，系统掌握经济、科技、管理、法律等专业知识，切实增强行政管理能力，努力成为本职工作和把握全局的行家里手。积极推动机关干部投身创业，在经济建设实践中增强服务意识、创新发展思路，提高驾驭市场经济能力，不断创造新的业绩。

　　各位代表，美好的发展前景令人振奋，艰巨的建设任务催人奋进。让我们以邓小平理论和三个代表重要思想为指导，全面贯彻党的十六大和十六届三中全会精神，更加紧密团结在以胡锦涛同志为总书记的党中央周围，在中共连云港市委的领导下，团结带领全市人民，解放思想、实事求是、与时俱进，为全面融入江苏两个率先大局，实现连云港快速崛起而努力奋斗。
